# Functions of Traditional Chinese Medicine Combined with Recombinant Human Interferon *α*2b in Cervical Intraepithelial Neoplasias Patients

**DOI:** 10.1155/2021/6881720

**Published:** 2021-12-09

**Authors:** Wei Ding, Xiaoyan Li, Baojun Ji, Zhenna Wang

**Affiliations:** ^1^Department of Ultrasound, People's Hospital of Rizhao, Rizhao 276800, China; ^2^Department of Blood Transfusion, Weifang People's Hospital, Weifang 261041, China; ^3^Special Inspection Department, Qingdao Women and Children's Hospital, Qingdao 266000, China

## Abstract

Cervical cancer is a common malignant neoplasm in women, and its incidence is increasing year by year. This study explored the effects of traditional Chinese medicine combined with recombinant human interferon *α*2b in cervical cancer patients. 178 cervical intraepithelial neoplasias (CIN) combined with high-risk HPV-positive patients from June 2017 to August 2020 were divided into the study group (*n* = 89 cases) and the control group (*n* = 89 cases) by the random number table method. Patients in the control group were treated with recombinant human interferon *α*2b, and the study group was treated with traditional Chinese medicine (TCM) on the basis of the control group. After treatment, the recurrence rate in the study group was significantly decreased while the human papillomavirus (HPV) negative conversion rate was significantly increased. 3 months after treatment, the TCM symptom scores in the study group were lower than in the control group. Moreover, serum levels of inflammatory factors decreased in both groups, and the decrease was more significant in the study group. After treatment, the ultrasound parameters were significantly decreased in the study group than in the control group. In conclusion, traditional Chinese medicine combined with recombinant human interferon *α*2b in cervical cancer patients could effectively improve the negative conversion rate of HPV infection, the level of inflammatory factors, reduce the degree of cervical erosion, and enhance the immunity of patients with high safety and significantly improve the quality of life.

## 1. Introduction

Cervical cancer is the most common female malignant tumor disease nowadays, and in recent years, with the change in people's lifestyles, the incidence of this disease has been increasing year by year, which seriously affects the quality of life of female patients [1]. Human papillomavirus (HPV) is a cyclic double-stranded small DNA virus that is epitheliophilic and invades the cervical basal metaplasia epithelial cells and squamous epithelial transformation zone [2]. Cervical columnar intraepithelial neoplasia trauma provides a good environment for HPV to proliferate, and therefore, cervical columnar intraepithelial neoplasia is susceptible to HPV infection [3]. When cervical columnar intraepithelial neoplasia is combined with persistent HPV infection, especially with high-risk human papillomavirus (HR-HPV), it is highly susceptible to carcinogenesis [4]. The results of a related study [5] showed that the development of CIN in patients was mainly caused by high-risk HPV infection and was also an important cause of cervical cancer. Therefore, enhancing the effective treatment of cervical HPV infection is important to reduce pain and improve the quality of life of patients.

Recombinant human interferon *α*2b is a commonly used antiviral drug that can effectively inhibit the replication of genetic material of the virus and enhance the immune system of patients, which can achieve better and shorter-term efficacy. However, the relapse rate is high after discontinuation of the drug, and long-term use of the drug may also affect the tolerance and compliance of patients [6]. In Chinese medicine, cervical HPV infection is classified as “leukorrhea” and “multicolored vaginal discharge,” which should be treated by invigorating the spleen, removing dampness, and invigorating the spleen-stomach and replenishing qi [7].

In this study, the Buqi Qushi Jiedu decoction used was a self-prepared decoction, which could effectively remove pathogens from the body, improve the overall state of the body, and mobilize specific and nonspecific immune functions. Therefore, in this study, recombinant human interferon *α*2b and Buqi Qushi Jiedu decoction were combined to treat patients with cervical intraepithelial neoplasia.

## 2. Materials and Methods

### 2.1. Clinical Data

A total of 178 patients with CIN admitted at the People's Hospital of Rizhao, Rizhao, Shandong, China, from June 2017 to August 2020 were selected as the study objects.

Inclusion criteria were as follows: Western medicine diagnosis conformed to the diagnostic criteria of CIN established by the American Society for Colposcopy and Cervical Pathology (ASCCP) [8]; Chinese medicine diagnosis conformed to the diagnostic criteria of spleen deficiency and dampness excess in the Guidelines for Clinical Research on New Chinese Medicines [9]; pathological examination confirmed the diagnosis of patients with CIN grade I–II; HPV-DNA gene chip test for high-risk HPV; and those without hematologic disorders. Patients voluntarily participated in this study and signed the informed consent form.

Exclusion criteria were as follows: patients who had undergone physical or surgical treatment of the cervix within the last 3 months; patients with severe cardiac, hepatic, and renal insufficiency; patients with acute or subacute inflammation of the reproductive organs, such as vaginitis and pelvic inflammatory disease; and patients with allergy to drugs used in this study.

They were divided into study and control groups using the random number table method, with 89 cases in each group. The age of the study group was 21 to 38 years old, with a mean age of 28.73 ± 3.63 years; 32 were married and 57 were unmarried; and the duration of the disease was 0.5 to 2 years, with a mean duration of 0.91 ± 0.27 years. The average age of the control group was 20 to 36 years, with a mean age of 28.41 ± 3.42 years; 35 cases were married and 54 cases were unmarried; the disease duration was 0.8 to 2 years, with a mean disease duration of 1.03 ± 0.34 years. There was no statistically significant difference between the general data of the two groups (*P* > 0.05). The study was approved by the ethics committee of the People's Hospital of Rizhao, Rizhao, Shandong, China.

### 2.2. Therapeutic Method

**Figure 1:**
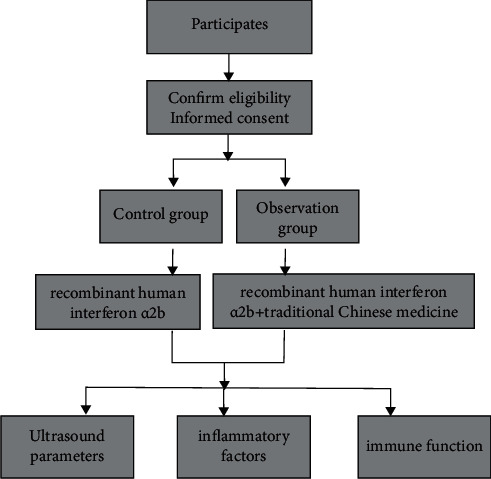
Flow diagram of therapeutic approach.

1. The control group was given a single recombinant human interferon treatment as follows: the patient was given recombinant human interferon on day 3 after the end of menstruation, the patient cleaned the vulva with a 0.5% potassium permanganate solution at bedtime, and a recombinant human interferon *α*2a suppository (State Drug Quantifier S20020103, Anhui Anke Biological Engineering, 100,000 IU/capsule) was disposed of in the posterior vaginal vault every other day. 1 capsule/time, 2∼3 times a week for 3 months.
2. On the basis of the control group, the study group was given Buqi Qushi Jiedu decoction for treatment. The formula was as follows: milkvetch root 15 g, sargentgloryvine stem 15 g, Chinese thorowax root 10 g, coix seed 15 g, amur cork-tree 10 g, flying squirrel's droppings 10 g, barbated skullcup herb 15 g, liquorice root 6 g, Chinese angelica 10 g, Indian bread 10 g, light-yellow sophora root 12 g. Decocted with water, 200 mL of the solution was taken in 1 dose/d, divided into two doses in the morning and evening. Patients stopped taking the medicine during menstruation, and the treatment was continued for 3 months. Patients in both groups were instructed to strictly prohibit raw, cold, spicy, and greasy food during treatment, to eat a light diet, to strictly prohibit alcohol and carbonated beverages, to prohibit smoking, to develop good habits of early to bed and early to rise, to maintain an optimistic attitude to participate in treatment, and to refrain from sexual intercourse during treatment to avoid infections. If discomfort occurs, the patient should promptly inform the medical staff for effective treatment. Patients should strictly follow the doctor's instructions during the treatment, and it is strictly forbidden to change the dosage or stop the medication without permission. To minimize errors, patients should not use other medications during treatment.  Figure 1 shows the flow diagram of the therapeutic approach used in this study.

### 2.3. Observation Indicators and Criteria

HPV negative conversion and HPV recurrence during follow-up were compared between the two groups. Cervical HPV exfoliated cells were collected, smears were performed, and then pap staining. An HPV negative conversion rate was detected according to HPV-DNA hybridization. HPV was detected by the PCR fluorescence method; the number of cases with negative conversion/total number of cases × 100% = HPV conversion rate [10]. Patients with negative HPV were followed up for 6 months to observe the HPV-positive condition, and the recurrence rate was calculated.

To compare the TCM symptom scores of the two groups, the symptom scores were evaluated according to the Guidelines for New Chinese Medicines [9] for the 2 groups before and 3 months after treatment for urinary frequency and urgency, leucorrhea and mucus, menstrual irregularity, pelvic heaviness, and dysmenorrhea, and each item was evaluated using a score of 0 to 3. The lower the score, the better the treatment outcomes.

The incidence of adverse reactions during treatment was compared between the two groups of patients. The lower the incidence of adverse reactions, the higher the safety of clinical treatment.

The levels of inflammatory factors before and after treatment were compared between the two groups. 3 mL of fasting venous blood was collected on day 1, 30 days, 60 days, and 90 days of treatment in both groups, and the levels of TNF-*α* and IL-2, 6, and 10 were measured by using an enzyme-linked immunosorbent assay.

The changes of immune function indexes in the 2 groups before and after treatment were detected. T cell lymphocyte subsets of CD3+, CD4+, and CD8+ were detected by flow cytometry.

Ultrasound parameters were compared between the two groups before and after treatment. TechnosDU8 (Prima Brands, Italy) with SonoVue (Bracco, Italy) contrast agent was used, and the probe frequency was 2.0–9.0 MHz. The uterus and the appendages of all patients were examined by two-dimensional ultrasound to determine the scanning surface and fix it. Then, switch to the contrast imaging mode and start the timer (3 min) at the same time as the contrast agent injection to observe the changes of blood perfusion and echo intensity. For the regions of interest in the scanning results, the relevant parameters were recorded: development starting time (T1), filling time (T2), peak time (T3), max intensity, area under the curve (area), rising slope (SLOPE1), fast descending slope (SLOpe2), and slow descending slope (SLOpe3).

Ultrasound parameters before and after treatment were compared between the two groups of patients. The ultrasound imaging machine used was TechnosDU8 (Esaote, Italy), the contrast agent was SonoVue (Bracco, Italy), and the probe frequency was 2.0∼9.0 MHz. The uterus and its adnexa of all patients were first scanned with 2D ultrasound, and the scanning surface was determined and fixed. Then, the imaging mode was switched to contrast imaging mode, and a timer (3 min) was started while the contrast agent was injected to observe the blood perfusion and echo intensity changes. For the area of interest in the scan results, the relevant parameters were recorded: start of contrast time (T1), filling time (T2), time to peak (T3), maximum intensity (max intensity), area under the curve (area), rising slope (slope1), fast falling slope (slope2), and slow falling slope (slope3).

The pain level and quality of life were compared between the two groups of patients before and after treatment. Pain level was measured by VAS score [11]; the VAS score was used to evaluate the pain level of the lower abdomen before and after treatment in both groups, with a total score of 0 to 10, and higher scores represented higher pain levels. Quality of life was evaluated by the quality-of-life scale [12] (SF-36 scale): the quality of life was evaluated by the SF-36 scale before and after treatment in both groups, and the total score of this scale was 0 to 100, and the higher the score, the better the quality of life of the patients.

### 2.4. Statistical Analysis

SPSS 20.0 software was used for statistical analysis, and the counting data were expressed as percentages. The *χ*2 test was used, and the measurement data were expressed as ‾*x* ± *s*. The *t*-test was used, and *P* < 0.05 was considered a statistically significant difference.

## 3. Results

### 3.1. Comparison of HPV Conversion Rate and Recurrence Rate after Treatment between the Two Groups

**Figure 2:**
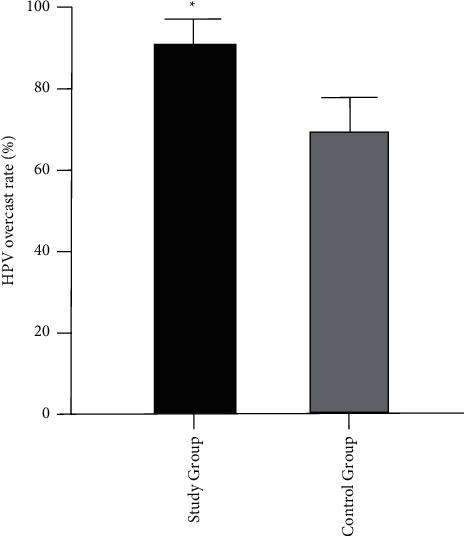
HPV conversion rate after treatment in both groups (*∗P* < 0.05).

**Figure 3:**
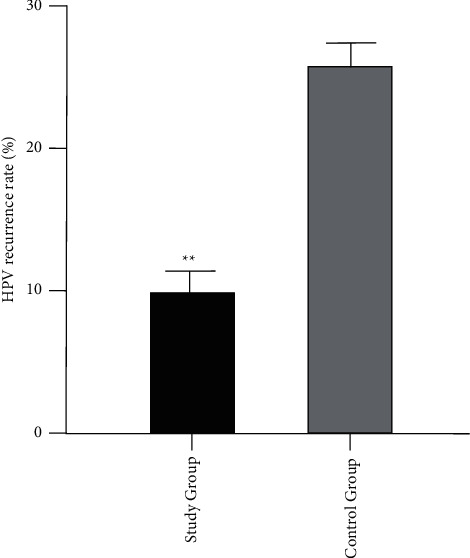
HPV recurrence rate after follow-up in both groups (^*∗∗*^*P* < 0.01).

There were 81 cases of HPV conversion in the study group, with a negative conversion rate of 91.01%, and 62 cases of HPV conversion in the control group, with a conversion rate of 69.66%. There were 8 cases of HPV recurrence in the study group, with a recurrence rate of 9.88%; 16 cases of HPV recurrence in the control group, with a recurrence rate of 25.81%. The difference between the data of both groups was statistically significant (*P* < 0.05, Figure 2 and 3).

### 3.2. Comparison of TCM Symptom Scores between the Two Groups

The TCM symptom scores of both groups 3 months after treatment were lower than those before treatment (*P* < 0.05); the scores of urinary frequency and urgency, leucorrhea and mucus, irregular menstruation, pelvic heaviness, and dysmenorrhea were lower than those of the control group 3 months after treatment in the study group (*P* < 0.05, Table 1).

### 3.3. Comparison of the Levels of Inflammatory Factors before and after Treatment between the Two Groups

**Figure 4:**
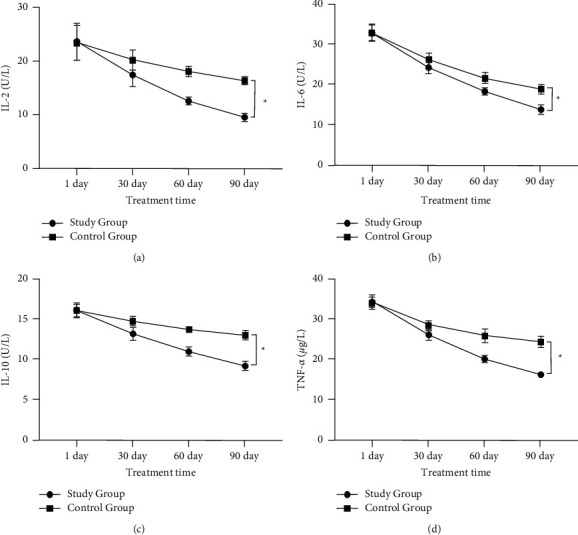
Comparison of inflammatory factor levels before and after treatment in two groups. (a) The comparison of IL-2 levels before and after treatment in two groups; (b) the comparison of IL-6 levels before and after treatment in two groups; (c) the comparison of IL-10 levels before and after treatment in two groups; (d) the comparison of TNF-*α* levels before and after treatment in two groups. ^*∗*^*P* < 0.05.

On the first day of treatment, there was no statistically significant difference in the levels of serum IL-2, IL-6, IL-10, and TNF-*α* between the two groups (*P* > 0.05), and the levels of IL-2, IL-6, IL-10, and TNF-*α* in both groups showed a decreasing trend with the extension of treatment time. After 90 days of treatment, the decrease of each index was more significant in the study group compared to the control group (*P* < 0.05, Figure 4).

### 3.4. Comparison of Ultrasound Parameters of Cervical Lesions before and after Treatment

The differences in max intensity, area, and slope1 between the two groups before treatment were not statistically significant; after treatment, max intensity, area, and slope1 decreased, and the differences were statistically significant before and after treatment (*∗P* < 0.05), and the decrease was more significant in the study group than in the control group (^△^*P* < 0.05) (Table 2).

### 3.5. Comparison of Immune Function between the Two Groups before and after Treatment

**Figure 5:**
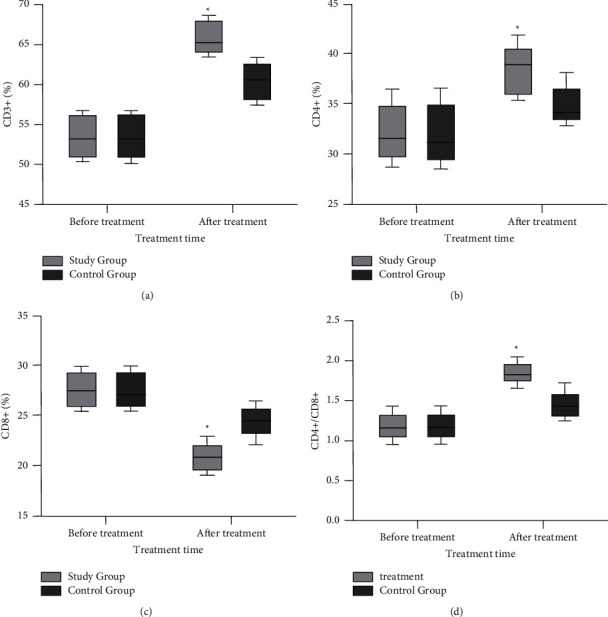
Comparison of immune function before and after treatment between the two groups. (a) The comparison of CD3+ levels before and after treatment between the two groups; (b) the comparison of CD4+ levels before and after treatment between the two groups; (c) the comparison of CD8+ levels before and after treatment between the two groups; (d) the comparison of CD4+/CD8+ levels before and after treatment between the two groups. ^*∗*^*P* < 0.05.

Before treatment, there was no statistically significant difference in the levels of each index of immune function between the two groups (*P* > 0.05). After treatment, the levels of CD3+, CD4+, and CD4+/CD8+ in the study group were significantly increased than those in the control group, while the CD8+ level in the study group was significantly decreased than that in the control group (*P* < 0.05, Figure 5).

### 3.6. Comparison of Pain Level and Quality of Life between the Two Groups before and after Treatment

Before treatment, there was no statistically significant difference in VAS scores and SF-36 scores between the two groups (*P* > 0.05). After treatment, VAS scores decreased in both groups, and the decrease was more significant in the study group than in the control group (*P* < 0.05). After treatment, SF-36 scores increased in both groups, and the increase was more significant in the study group than in the control group (*P* < 0.05, Table 3).

### 3.7. Comparison of the Incidence of Adverse Reactions after Treatment between the Two Groups

There was no statistically significant difference in the incidence of adverse reactions between the two groups (*P* > 0.05, Table 4).

## 4. Discussion

HPV is a double-stranded cyclic DNA virus divided into high- and low-risk types according to the risk of tumorigenesis. HPV infection in humans often develops into CIN, and high-risk HPV is closely associated with the development of cervical cancer [13]. According to the tissue origin, CIN is classified into three grades: I, II, and III, and the degree of involvement of cervical epithelial cells deepens successively [14]. During the progressive development of CIN, regular HPV examination, early detection, and treatment are of great significance to reduce the incidence and mortality of CIN and even cervical cancer [15]. The number of female patients with HPV infection is currently increasing, and effective treatment usually includes surgery and medications. However, due to the generally low age of the patients, they may be nervous during the surgery as it may cause trauma to the cervix's surface, which affects the quality of life of the patients and is especially unfavorable for unmarried and infertile women [16].

Recombinant human interferon, which is a protein in nature, can bind to interferon receptors on the surface of affected tissues and exert antiviral effects, which can interfere with viral replication, thereby inhibiting viral synthesis, enhancing the phagocytosis of macrophages to strengthen the immune function of patients, regulate progesterone levels in the body, and improve the internal environment of the patient's vagina [17].

Recombinant human interferon *α*2b is a new interferon agent that is mostly used clinically to treat HPV infection [18]. Recombinant human interferon can be placed in the patient's vagina, and local administration will not cause damage to the patient's organism, and the application is simple and convenient, which is easily accepted by patients. *α*2b can improve the killing capacity of the body's lymphocytes, enhance the phagocytosis of macrophages, reduce cervical secretions during patient treatment, improve vaginal cleanliness, enhance the body's immunity, reduce clinical adverse effects, and improve the ability to fight against viruses [19]. According to Sen et al. [20, 21], *α*2b can exert an induction effect on antiviral proteins, enhance phagocytosis of macrophages, stimulate lymphocytes, inhibit viral protein synthesis, and thus inhibit HPV replication and transcription, which is important for the removal of HPV virus.

In Chinese medicine, HPV is categorized as “morbid vaginal discharge” or “parti-colored vaginal discharge” according to the patient's clinical manifestations. According to recent research, cervical HPV infection is mainly due to the channel imbalance and the conception channel irregularity of the leukorrhea and the invasion of dampness, which causes damage to the cells surrounding the cervix, resulting in changes in the odor, color, and state of vaginal secretions and eventually leading to the development of the disease [22, 23]. In clinical practice, the main treatments are clearing heat, removing toxicity, invigorating the spleen, and eliminating dampness. In this study, the scores of urinary frequency and urgency, leucorrhea and mucus, irregular menstruation, pelvic heaviness, and dysmenorrhea in the study group were lower than those in the control group after treatment (*P* < 0.05), indicating that Buqi Qushi Jiedu decoction combined with recombinant human interferon *α*2b can effectively improve the symptom score of patients with cervical HPV infection and facilitate their recovery. In this formula, the combination of milkvetch root, light-yellow sophora root, Indian bread, and liquorice root can achieve the effects of invigorating the spleen and eliminating dampness; the combination of sargentgloryvine stem, Chinese thorowax root, and amur cork-tree can achieve the effect of clearing heat and removing toxicity; the combination of barbated skullcup herb, flying squirrel's droppings, and Chinese angelica has the effect of promoting blood circulation for removing blood stasis; and Coix seed has anti-inflammatory and antitumor functions and can enhance the immune functions of the body. The rational combination of the abovementioned drugs has the ability to clear heat and remove toxicity, invigorate the spleen and stomach, and replenish qi [24–26].

In this study, serum TNF-*α*, IL-2, 6, and 10 were lower in the study group than in the control group after treatment (*P* < 0.05), suggesting that Buqi Qushi Jiedu decoction could significantly improve the levels of inflammatory factors in patients with cervical HPV infection and control disease development. Studies [27–29] have shown that the main way for human immune cells to exert their biological activity is through their own secreted cytokines, and inflammatory factors such as IL-2, IL-6, IL-10, and TNF-*α* have essential roles. IL-2 plays an active role in antitumor and antipathogen immunity. Il-6 can regulate the growth and differentiation of various cells, regulate immune response, acute phase response, and hematopoietic function, and plays an essential role in the anti-infection immune response of the body. IL-10 has an active role in enhancing the infection sensitivity of immune cells. TNF-*α* is a proinflammatory cytokine expressed in the development of tissue inflammation response. We also observed that the CD3+ and CD4+ levels and CD4+/CD8+ ratios were significantly higher and CD8+ levels were significantly lower in both groups after treatment, and the changes in these indexes were more significant in the study group (*P* < 0.05), and the HPV negative conversion rate was significantly higher in the study group than in the control group, and the recurrence rate was significantly lower than in the control group. The combination of Buqi Qushi Jiedu decoction with recombinant human interferon *α*2b suppository had a positive effect on HPV clearance and improvement of immune function in patients, which could effectively prevent and control the occurrence of cervical inflammatory reactions, improve the HPV conversion rate and histological efficacy of cervical lesions, and reduce the long-term recurrence rate of patients with CIN combined with HR-HPV.

In this study, the combination of Buqi Qushi Jiedu decoction with recombinant human interferon *α*2b did not increase the incidence of adverse effects and helped improve patients' treatment tolerance and compliance. Ultrasonography can show blood perfusion at the capillary level and can monitor the tissue microvascular environment [30]. The results of this study showed that the maximum intensity, area, and slope1 of both groups gradually decreased as the condition improved, and the decrease was more significant in the study group (*P* < 0.05). The max intensity indicates the amount of contrast medium when the local vessels are filled with the most contrast medium, reflecting the maximum blood volume; slope1 represents the local perfusion rate, which becomes slower as the condition continues to improve [31, 32]. Therefore, among the parameters of ultrasonography, max intensity, area, and slope1 have high clinical significance and can provide a clinical basis for early screening of CIN combined with HR-HPV. The follow-up time of this study was limited, and the long-term effective rate of combination therapy could be analyzed by increasing the sample size and extending the follow-up time.

In conclusion, the combination of Buqi Qushi Jiedu decoction with recombinant human interferon *α*2b could make the qi and blood flow smooth in patients with CIN combined with HR-HPV, and the clinical effects were favorable. It could significantly improve the HPV conversion rate, reduce the distant recurrence rate, and lower the TCM symptom score. It could reduce the level of inflammatory factors and improve the immunity of the body with high safety.

## Figures and Tables

**Table 1:** Comparison of TCM symptom scores between the two groups ($\bar{x}$ ± *s*).

Symptoms	Study group	Control group	*t*	*P* value
*Urinary frequency and urgency*				
Before treatment	2.51 ± 0.32	2.53 ± 0.34	0.227	>0.05
3 months after treatment	0.65 ± 0.14	1.76 ± 0.22	4.652	<0.05

*Leukorrhea and mucus*				
Before treatment	2.57 ± 0.26	2.54 ± 0.31	0.346	>0.05
3 months after treatment	0.71 ± 0.18	1.63 ± 0.27	10.371	<0.05

*Menstrual irregularities*				
Before treatment	2.43 ± 0.48	2.45 ± 0.52	0.732	>0.05
3 months after treatment	0.96 ± 0.11	1.72 ± 0.34	7.611	<0.05

*Pelvic heaviness*				
Before treatment	2.46 ± 0.34	2.44 ± 0.35	0.546	>0.05
3 months after treatment	0.83 ± 0.16	1.68 ± 0.23	6.230	<0.05

*Dysmenorrhea*				
Before treatment	2.37 ± 0.58	2.34 ± 0.56	0.934	>0.05
3 months after treatment	0.74 ± 0.17	1.65 ± 0.29	12.456	<0.05

**Table 2:** Comparison of ultrasound parameters of cervical lesions before and after treatment.

Index	Study group (*n* = 89)		Control group (*n* = 89)	
	Before treatment	After treatment	Before treatment	After treatment
T1(s)	13.673	10.357	13.224	12.253
T2(s)	15.894	16.861	15.978	16.249
T3(s)	33.146	27.462	33.317	30.175
Max intensity(dB)	213.678	116.894^*∗*^^△^	215.266	131.246^*∗*^
Area	18237.662	13798.519^*∗*^^△^	18368.143	16037.030^*∗*^
Slope1	9.774	5.792^*∗*^^△^	9.681	7.234^*∗*^
Slope2	−2.253	−2.015	−2.287	−2.071
Slope3	−0.414	−0.322	−0.426	−0.353

^*∗*^ *P* < 0.05: before treatment vs. after treatment. ^△^*P* < 0.05: study group vs. control group.

**Table 3:** Comparison of pain levels and quality of life before and after treatment between two groups of patients.

Index		Study group	Control group	*t*	*P* value
VAS score	Before treatment	6.23 ± 1.67	6.17 ± 1.53	0.217	>0.05
	After treatment	1.96 ± 0.52	3.68 ± 0.93	6.431	<0.05

SF-36 score	Before treatment	44.23 ± 6.74	43.67 ± 6.52	0.933	>0.05
	Before treatment	78.63 ± 9.56	61.71 ± 8.67	7.249	<0.05

**Table 4:** Comparison of the incidence of adverse reactions after treatment in the two groups (*n* (%)).

Group	*n*	Increased secretion	Vaginal itching	Vulvar pain	Nausea and vomiting	Liver and kidney abnormalities
Study group	89	4(4.5)	1(1.1)	2(2.2)	2(2.2)	1(1.1)
Control group	89	7(7.9)	2(2.2)	4(4.5)	5(5.6)	3(3.4)
*X* ^2^						0.230
*P* value						0.994

## Data Availability

The data used to support the findings of this study are available from the corresponding author upon request.
